# Mockingbird Morphing Music: Structured Transitions in a Complex Bird Song

**DOI:** 10.3389/fpsyg.2021.630115

**Published:** 2021-05-04

**Authors:** Tina C. Roeske, David Rothenberg, David E. Gammon

**Affiliations:** ^1^Max Planck Institute for Empirical Aesthetics, Max Planck Society, Frankfurt, Germany; ^2^New Jersey Institute of Technology, Newark, NJ, United States; ^3^Elon University, Elon, NC, United States

**Keywords:** birdsong analysis, musicality, mockingbird, sonograms, song complexity

## Abstract

The song of the northern mockingbird, *Mimus polyglottos*, is notable for its extensive length and inclusion of numerous imitations of several common North American bird species. Because of its complexity, it is not widely studied by birdsong scientists. When they do study it, the specific imitations are often noted, and the total number of varying phrases. What is rarely noted is the systematic way the bird changes from one syllable to the next, often with a subtle transition where one sound is gradually transformed into a related sound, revealing an audible and specific compositional mode. It resembles a common strategy in human composing, which can be described as variation of a theme. In this paper, we present our initial attempts to describe the specific compositional rules behind the mockingbird song, focusing on the way the bird transitions from one syllable type to the next. We find that more often than chance, syllables before and after the transition are spectrally related, i.e., transitions are gradual, which we describe as morphing. In our paper, we categorize four common modes of morphing: timbre change, pitch change, squeeze (shortening in time), and stretch (lengthening in time). This is the first time such transition rules in any complex birdsong have been specifically articulated.

## Introduction

**FIGURE 1:**
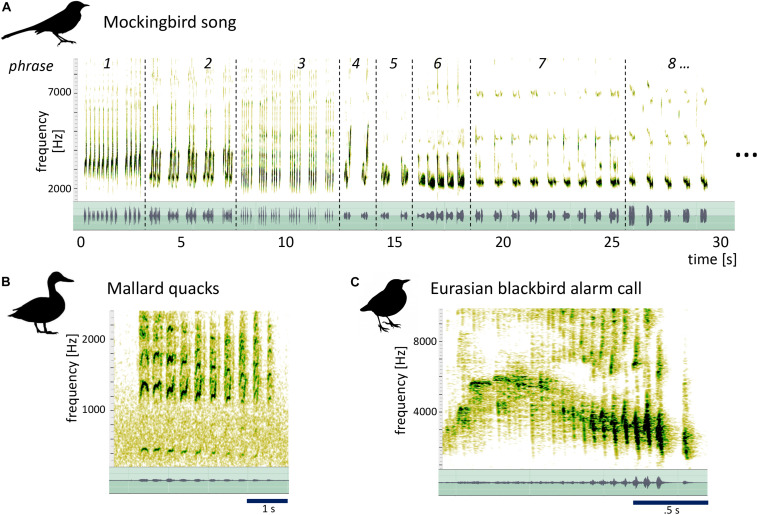
Spectrogram (top) and waveform (bottom, green) of mockingbird song, illustrating its hierarchical structure, and mallard and blackbird calls for comparison. **(A)** 14 s of male mockingbird song. Dotted lines mark phrase transitions. Both spectrogram and waveform illustrate the organization of Birdsong material in distinct phrases of repeated syllables (e.g., phrase 3) or syllable groups (e.g., phrase 1). **(B)** A series of mallard quacks (XC613266, recorded by Jack Berteau in the Vendée, France) and **(C)** a blackbird’s alarm call (XC622587, recorded by Pierre Foulquier in Le Mans, France) that both contain a specific kind of acoustic morphing: one repeated note/syllable gradually slows down and descends in pitch (after an initial rise for the blackbird call). We argue here that the variegated morphing modes observed across mockingbirds’ phrase transitions are distinct from these cases of stereotyped within-phrase morphing, less easily explained by muscular constraints, and surprisingly similar to certain strategies in music. The mallard quacks and the blackbird call in Figure 1 were obtained from the Xeno-Canto database. The mallard was recorded by Jack Berteau in the Vendée, France (recording XC613266) and the blackbird by Pierre Foulquier in Le Mans, France (recording XC622587).

Among songbirds, the mockingbird has an extraordinarily variegated song that is much more complex than most species’ songs. Mockingbirds have enormous repertoires of song elements (Derrickson, 1987, 1988; Kershenbaum et al., 2015) which are arranged in a particular way: individual “syllables” (which can be a single sound, as in phrase 3 in Figure 1A, or a small group of sounds, as in phrase 1) are repeated to form short phrases, which, in turn, are strung up into long songs that can go on for minutes (Figure 1A). As indicated by their name, mockingbirds are also famous for their ability to mimic the sounds of other species or their acoustic environment (Whittle, 1922; Laskey, 1944; Baylis, 1982; Farnsworth et al., 2011; Gammon, 2013; Gammon and Corsiglia, 2019).

A single audio file is included in the Supplementary Material which covers all the figures in this paper. We have also made a video that demonstrates the basic morphing techniques used by this species, compared with examples of human music^1^. A second video shows a longer clip of a single mockingbird singing^2^, revealing a longer series of the transitions we categorize in the paper.

While all of this mimicry and complexity presumably evolved for bird ears, it has long fascinated humans as well, and even inspired us to create our own aesthetic productions from poetry to music (examples reach from the lullaby “Hush little baby” and the “Out of the Cradle Endlessly Rocking” of Walt Whitman to Suzanne Collins’s dystopian young adult novel *Mockingjay*; Rothenberg, 2005). When humans listen to mockingbird song, they often notice that successive song phrases are acoustically related, similar to what musicians call variations of the same theme. In other words, the transition from one mockingbird phrase to the next often represents a gradual acoustic shift, keeping most features the same, but changing one or more acoustic features, such as pitch or tempo. We call this phenomenon *morphing* (note that we use this term here to describe spectral relatedness between *individual notes*, not within continuous sound units). This morphing is easily grasped by human listeners, even to the untrained ears of non-ornithologists who might recognize less of the mimicry. We suspect that one major reason mockingbird song so fascinates humans lies in the fact that morphing is also a central quality in human music.

Morphing may be a prominent feature of mockingbird song, but it has rarely been investigated. Instead, studies on the structure of mockingbird song have focused on determining repertoire size (Howard, 1974; Derrickson, 1987; Kershenbaum et al., 2015), overall temporal and frequency characteristics (Wildenthal, 1965), seasonal differences in song structure (Logan and Fulk, 1984; Gammon, 2014), and particularly the extensive vocal mimicry (Gammon and Altizer, 2011; Gammon, 2014; Gammon and Lyon, 2017; Gammon and Corsiglia, 2019). For other songbird species, acoustic relationships between adjacent or near-adjacent notes have rarely been a research focus either—despite their saliency in some species. Among the few studies that touch upon acoustic relationships within sequences are the descriptions of themes and variations in the song of the Bachman’s sparrow (Hartshorne, 1973), general claims that such sound structures are common in birds (Baptista and Keister, 2005) and evidence for mathematical properties of thrush nightingale rhythm (namely, multifractality) that may point, albeit indirectly, to variations as well as gradual modifications in rhythm (Roeske et al., 2018). Transitional relationships between adjacent (or near-adjacent) elements have been studied extensively in terms of *syntax*—i.e., transitional probabilities in note sequences (e.g., Kershenbaum et al., 2014; review by Berwick et al., 2011) or focusing on memory (Hultsch and Todt, 1989)—but not in terms of the spectral properties or similarity between those notes. We cannot rule out, based on our analysis, that the mockingbirds’ acoustic morphing might reflect a functionless byproduct of how song phrases are stored in the brain and produced by the syrinx. However, when compared to acoustic morphing observed in many birds’ calls (see examples in Figures 1B,C) that are seamlessly explained by motor constraints (muscle fatigue and constraints on breathing), the modes of morphing we observe in mockingbird song are considerably more variegated, span longer sequences, are less stereotyped, and often operate not on repetitive same-syllable trains but higher-order groups, with morphed syllables often interspersed by spectrally very different syllables (see examples in Figures 5B, 6B, 7C). Given the variability and prominence of acoustic morphing in mockingbird song transitions, as well as the importance we attach to morphing-like transition strategies in our own music, we find it fruitful to examine mockingbird song through a more musicological lens. We argue here that the variegated modes of mockingbird phrase-transition morphing are closer to those musical strategies than the stereotyped syllable morphing in mallard quacks, blackbird warning calls, or the like (see Figures 1B,C), which is more in line with efficiency-based explanations.

Why has morphing not been studied in mockingbirds? Perhaps putting animal communication too close to a human behavior that centrally aims at *subjective* pleasantness or interestingness has been too suspicious for a science like biology that strives for objectivity. Interestingly, however, Charles Darwin himself wrote of species-specific qualities in birdsong as representing an “aesthetic sense,” (Darwin, 1871) apparently accepting birds’ subjective judgments as potentially shaping singing behavior.

We are far from anthropocentrically claiming that the same evolutionary, cognitive, and emotional drivers underlie mockingbird song that have shaped human music. Nevertheless, the *subjective* qualities of birdsong *are* a central ingredient of the sexual selection processes that drive birdsong evolution (Prum, 2017): Being subjectively intrigued by a song is probably a key step for a female in considering the singer as a mate, but the subjective side has largely been ignored in research. We suspect that female mockingbirds pay close attention to male song, given that a male’s song output closely correlates with the fertility of his mate (Merritt, 1985; Wright, 2014), given that a male triples his song output if his mate is removed (Logan and Hyatt, 1991), and given that copulations are always accompanied by male song production (Merritt, 1985; Gammon, unpublished data). Existing research on female preference for male song properties (like particular vocal elements or repertoire size) does, of course, assume the same—that females attend to male song features. However, the main perspective of this research has usually been that song aspects which trigger female readiness for mating—rather like in a stimulus-response loop—are probably honest signals for male quality, and the females’ attention for it is therefore adaptive. The difference in our approach is that we simply turned our attention to sounds that are subjectively intriguing (to us), without having to assume honest signaling, or adaptivity. Our focus is not on how singing may stand for something else (i.e., male quality), but on how sounds raise human (and perhaps bird) listeners’ interest and attention. And given that birdsong is complex, variable, and intriguing across species boundaries—instead of just being a continuous repetition of the one “sexiest” sound—we think that understanding what acoustic structures in particular are able to intrigue listeners is essential for understanding birdsong evolution (Roeske et al., 2018). Of course, we are aware that this hypothesis rests on assumptions that are far from proven: a mockingbird’s perception of note sequences might differ from that of humans. What we perceive as morphs may not be morphs for them (and vice versa). There is a chance that the birds are perceptually oblivious to morphing, and future research on morphing perception is needed to clarify its potential roles and evolution.

We do not yet have any tools to investigate subjective qualities in animals (or humans) directly. But we can systematically investigate song structures that are likely candidates for transporting relevant subjective qualities, because they are known to play such a role in our music. To understand where parallels between human and animals signaling exist, how far they extend, and where the similarity breaks down, a careful description of the possibly similar phenomenon is essential.

In this exploratory analysis of mockingbird phrase transitions, we explore morphing as an acoustic property that may potentially be salient to the birds and making their songs more effective. To provide some context to our description of morphing, we start by giving an overview over *other* known structural aspects of mockingbird song.

Next, we give a detailed *qualitative* description of the phenomenon of morphing, from a musicology-inspired perspective: This first description is based on subjectively listening to adjacent phrases, as well as visual inspection of spectrograms. We identify four different modes of morphing: (1) Pitch-based morphing, (2) timbre-based morphing, (3) temporal stretching, and (4) temporal squeezing. We relate these four modes to human musical examples with similar properties. Note that these modes are not exclusive; they can be overlapping, and alternative classification systems are conceivable. We therefore do not consider this qualitative description as an objective, exhaustive classification system, but rather as a heuristic. As such, however, it leads to specific predictions that can be objectively tested: If morphing indeed *exists* in mockingbirds song—i.e., if it is more than just a pattern suggested to us by our biased human auditory system—we expect

• Instances of morphing to be common throughout the song.
• The average similarity between adjacent phrase pairs to be higher than between random, distant pairs of phrases, and this effect should be measurable for different acoustic features that capture pitch, timbre, and temporal structure of syllables.

Finally, we present quantitative (albeit exploratory) evidence to test these two predictions: Using sample songs from four birds, we quantify their use of the different modes of morphing. Analyzing acoustic similarity between phrases, we test whether morphing is more common in their songs than expected by chance.

Ultimately, our study aims to understand better what kinds of mockingbird sound sequences elicit their listeners’ subjective interest. We describe here one such candidate structure, the morphing of phrases into new, but acoustically related phrases. Our results can serve as a basis for future studies that test experimentally whether the presence or absence of morphing in song indeed affects its listeners’ reactions.

### Background: the Structure of Mockingbird Song

Mockingbird song is repetitive; mockingbirds usually repeat each syllable three to five or more times before switching to a new type of syllable (Wildenthal, 1965). Each syllable can be a single note or a cluster of notes. Repeated syllables are normally separated by tiny breaths (“minibreaths”), which physiologically constrains the rate of syllable delivery to some extent (Zollinger and Suthers, 2004). Wildenthal (1965) has provided a careful description of general temporal and frequency properties of mockingbird song, from number of syllables per phrase and syllable grouping to overall frequency range. She provides mean values or distributions over entire song performances, but did not focus on the dynamic succession of elements with specific acoustic features.

### Mimicry

Mockingbirds are particularly famous for their ability to mimic. They will mimic the sounds of birds and other environmental sounds, as long as these sounds are acoustically similar to the typical acoustic feature range of mockingbird vocalizations (Gammon, 2013). Studies of model selection show, for instance, they mimic blue jay calls but not raven calls, American robin calls but not American robin songs, tree frogs but not bullfrogs (Gammon, 2013; Gammon and Corsiglia, 2019). About half of all song utterances are mimetic (Gammon, 2014), but it remains unclear whether mimetic songs are normally learned from heterospecific models or conspecifics already singing the mimetic song. The mockingbirds’ inclination to imitate a model is thus much less restricted than that of most other species: Most songbirds only learn to imitate the songs of conspecifics, sometimes even just their fathers (Grant and Grant, 1996).

### Repertoires and Sequential Progression of Song Elements

Overall repertoire sizes of mockingbirds are enormous, with individuals each producing hundreds of phrase types (Derrickson, 1987, 1988; Kershenbaum et al., 2015). After classifying thousands of song phrases from four birds, Derrickson (1987) showed that although mockingbirds produce hundreds of syllable types, the vast majority of syllable types are produced very rarely, with 25% of syllable types appearing just once in his sample. Similarly, most mimetic syllable types are also produced rarely (Gammon, 2013). Although most song-learning in mockingbirds takes place during the first year of life (Gammon and Tovsky, 2021), there is some evidence that mockingbirds continue to learn new syllable types throughout adulthood (Derrickson, 1987; Gammon, 2020).

How mockingbirds pick which syllable type from their repertoire to sing after completing phrase X, is poorly understood. Contrary to assertions from early studies (e.g., Wildenthal, 1965; Derrickson, 1987), mockingbirds do *not* sample randomly from their repertoires when ordering their sequences of song phrases (Gammon and Altizer, 2011; Kershenbaum et al., 2015). For example, the syllable types used in sequential phrases are often taxonomically related at a level several times higher than would be predicted by randomly ordering syllable types (Gammon and Altizer, 2011). These “taxonomic doubles” exist on multiple levels and are produced two to four times as often as chance would predict (Gammon and Altizer, 2011). Their function is unknown, but their production likely reflects the neural organization of song memories within the brain (Gammon and Altizer, 2011). For example, a bird might produce a sequence of taxonomically-related syllable types because the syllable types were all learned from the same model and might thus be stored in close proximity to each other, similar to the way human brains lump together songs that come from different tracks of the same music album.

Taxonomic doubles exist on multiple levels (Gammon and Altizer, 2011). A Type I taxonomic double consists of sequential syllable types that both represent mimicry of the same vocalization category of the same species, e.g., the two syllable types both classify as the “loud” song of the eastern bluebird (see Figure 6B). In contrast, a Type II taxonomic double consists of two sequential syllable types that classify as different vocalization categories of a mimicked species, e.g., a syllable classifies as an eastern bluebird “loud” song, followed by a syllable that classifies as an eastern bluebird “tuawee” call. The distinction is important for our paper because Type I taxonomic doubles are acoustically similar, whereas Type II taxonomic doubles are often acoustically dissimilar. Therefore, the production of Type I taxonomic doubles overlaps some with morphing. Just as taxonomic doubles likely reflect the neural organization of song memories, morphing might also reflect one song phrase triggering the neural release of an acoustically-similar song phrase.

## Materials and Methods

### Mockingbird Song Material

Mockingbird song material analyzed in this exploratory analysis is from five individual birds. Three of them were recorded in mid-spring by Dave Gammon, and two more were obtained from the publicly available birdsong database xeno-canto^3^ (see Table 1 for recording details).

**TABLE 1:** Recording details on the five birds analyzed in this study.

Bird ID	Recording length	phrases in quantitative analysis	Recording source	Time	Location
A	9:57 min	216	Dave Gammon	Mid-spring song	Elon, North Carolina
B	11:27 min	235	Dave Gammon	Mid-spring song	Elon, North Carolina
C	6:49 min	–	Dave Gammon	Mid-spring song	Elon, North Carolina
D	6:09 min	116	Paul Marvin, xeno-canto ID: 139965	Late spring song	Osceola, Florida
E	6:39 min	196	Richard E. Webster, xeno-canto ID: 321899	Late spring song	Cochese County, Arizona

In our analyses, we used these recordings as follows:

The *qualitative* analysis is based on bird A. Dave Gammon has listened to this particular bird for many years, and annotated the recordings by imitation/non-imitation.

For quantification of occurrence of the four morphing types (Figure 9), we used birds A, B (2-min excerpt), and C.

To quantify similarity between adjacent vs. distant phrases (see below), we used recordings of four birds, birds A, B, D, and E. We dropped bird C from this analysis due to inconsistent recording quality.

### Analysis

#### Segmentation of Mockingbird Song

Acoustic feature extraction from audio and segmentation of song into syllables was carried out in Matlab_R2018b (The MathWorks, Inc., Natick, MA, United States), using the package “Sound Analysis for Matlab” (by Sigal Saar). Amplitude envelopes were extracted in 10 ms time windows and steps of 1 ms. They were then normalized in time windows of 4 s, to minimize the influence of intensity fluctuations (e.g., due to changes in singing position) on the amplitude distance measures. Wiener entropy and frequency contours were extracted with the help of Sound Analysis Tools for Matlab from Sound Analysis Pro 2011 (Tchernichovski et al., 2000^4^). Frequency contours were based on mean frequency per ms, a pitch measure that assesses the center of the distribution of power across frequencies.

Segmentation was performed based on an amplitude threshold combined with a Wiener entropy threshold (as syllables were identified sounds that were *louder* than the amplitude threshold and had *lower* Wiener entropy than the Wiener entropy threshold). This procedure provided robust segmentation of all mockingbird recordings.

#### Qualitative Analysis of Morphing

Spectrograms were made with Martin Hairer’s program Amadeus Pro, which we use because it has great flexibility in adjusting the resolution and appearance of the spectrogram itself. This helps in visualizing aspects of the song that we hear and consider relevant for this analysis. The spectrograms were then coded and interpreted to further illustrate the “objectivity” of the morphing modes we identify, and to help with the statistical analysis that we then undertook.

We first described our auditory impression of morphing modes in the mockingbird song in a qualitative way, based on listening to recordings and visually inspecting spectrograms. The morphing modes we identified express the acoustic relationship between pairs of similar-sounding adjacent phrases (Figure 1A). We concentrated in this analysis on phrase transitions with salient similarity between the pre- and post-transition phrases, ignoring dissimilar transitions. We judged a phrase pair’s similarity/dissimilarity based on our auditory impressions and subjective saliency. In an iterative, but informal process, the authors converged on four modes of morphing that are named after and describe the *most clearly changed quality*. Science thrives on simplicity. At first, we heard 12 specific modes of morphing, then 10, then eight. We argued and wrestled, compared our listening skills: the naturalist, the analyst, the musician, and all decided we should reduce, reduce. So in this paper we emphasize four basic modes of morphing. We know these categories are oversimplified, and that almost every transition involves a mixture of more than one of these modes. We have quantified the frequency of the four modes of morphing using sample songs of the three birds A, B, and C.

#### Example Quantitative Analysis: Testing Whether Syllable Similarity Is Higher in Adjacent vs. Random Phrases

**FIGURE 2:**
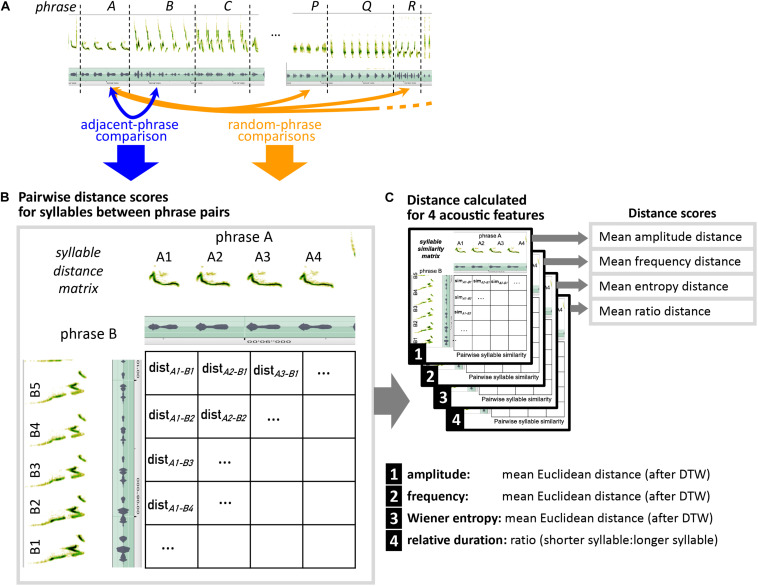
Schematic explaining how pairs of adjacent phrases vs. pairs of random, distant phrases are compared, to determine whether adjacent-phrase similarity is higher than expected by chance. **(A)** Every phrase (e.g., here phrase A) is compared to its following phrase (B), and to 10 different non-adjacent phrases from outside of a 10-phrase range around phrase A. **(B)** For each phrase pair, all syllables of the first song phrase are being compared to all syllables in the second phrase in a pairwise fashion. **(C)** Distance scores are calculated separately for five acoustic features. For each feature, the distance score of a phrase comparison is the mean from all syllable comparisons.

To provide a quantitative way of telling whether morphing—i.e., transitioning from one phrase of sound elements to a phrase of related sound elements—is a true feature of mockingbird song, as opposed to a human heuristic created by biases of our own auditory system, we investigated whether measurable acoustic similarity of *adjacent* phrases is higher than expected by chance. To this end, we compared pairs of adjacent phrases with random pairs of random, distant phrases (Figure 2A). The distant-phrase comparison serves as a proxy for what is expected by chance (i.e., if acoustic similarity is not an organizing principle of mockingbird song). “Comparing phrases” means we compared *acoustic features of individual syllables* in a pairwise fashion between both phrases (Figure 2B).

**FIGURE 3:**
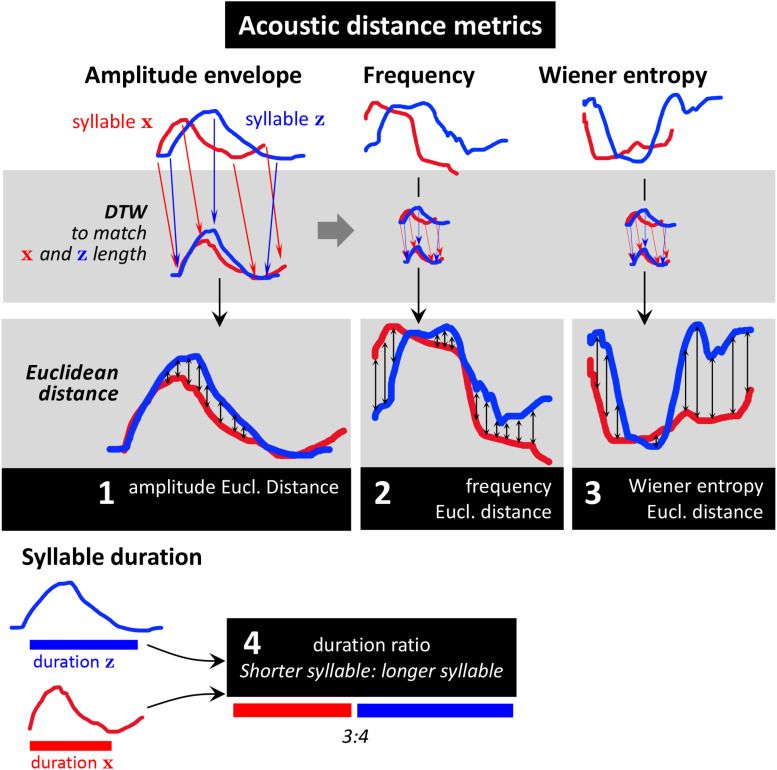
Distance metrics used in this study. The distance metrics were designed to reflect the different kinds of acoustic syllable similarities/differences obtained from qualitative analysis. Acoustic features captured by our analysis are amplitude envelope, frequency (a measure of pitch), Wiener entropy (a measure of timbre), and syllable duration. We performed dynamic time warping (DTW) on the two syllables’ amplitude envelopes (upper gray panel), using the same warping path on amplitude, frequency, and entropy contours. Then we calculated mean Euclidean distance between the two syllables for each feature (bottom gray panels). To describe differences in syllable duration, we calculated their duration ratio (shorter syllable: longer syllable; bottom).

To measure acoustic similarity, we chose four different metrics designed to reflect acoustic properties that play a role in the different perceived modes of morphing (Figures 2C, 3):

1. The amplitude envelope that captures overall syllable shape (e.g., reflecting whether it is a complex, two-note syllable or a uniform whistle)
2. Syllable frequency which captures the pitch contour
3. Wiener entropy which captures timbre (it is high in noisy and low in tone-like sounds)
4. Syllable duration.

To compare a syllable pair’s millisecond-wise time series of amplitude envelope, frequency, and Wiener entropy, we first applied dynamic time-warping (DTW) of the two amplitude envelopes (Figure 3, top left). We then calculated *mean Euclidean distance* between the time-warped syllable features (Figure 3, middle). We compared syllable durations (which are single values, no time series) by calculating their ratio (shorter duration: longer duration; see Figure 7, bottom). For each pair of phrases, a distance score was calculated for each feature by averaging across all pairwise-syllable comparisons. For example, to make the phrase comparison illustrated in Figure 2B, the distance score would be calculated by averaging 20 bioacoustic measurements.

We expect that morphing affects Euclidean distance of these features in specific ways (see also Table 2): squeezing and stretching will lead to different durations before and after the phrase transition. This will result in low duration ratios (the more different the durations, the lower their ratio). At the same time, Euclidean distances in amplitude, frequency, and entropy will be lower than between the syllables of random phrase pairs. Further, we expect morphing modes pitch change and timbre change to result in low Euclidean distances in all features *but* frequency and Wiener entropy, respectively. Finally, we expect *all* morphing modes to result in lower Euclidean distance between amplitude envelopes than between random pairs of phrases.

**TABLE 2:** How the four morphing modes would affect the expected syllable similarity between *adjacent* phrases.

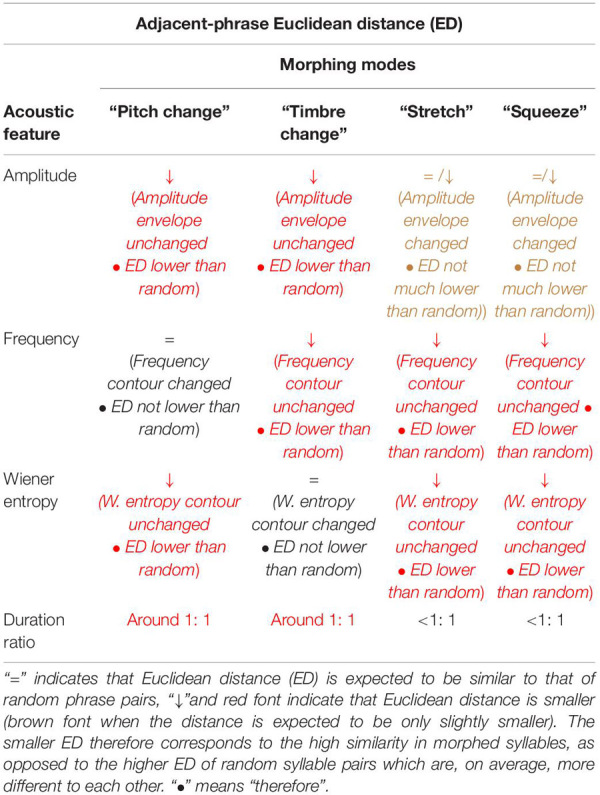				

### Statistics

To assess significance of the difference between Euclidean distances of adjacent vs. distant phrases, we performed *t*-tests and applied Bonferroni correction for multiple testing.

## Results

### Qualitative Analysis: Four Categories of Morphing

We present here the four modes of morphing that resulted from auditory evaluation of a 10-min song performance of one male bird, visual inspection of the spectrogram, and several rounds of informal communication on possible ways to cluster our subjective listening and visual impressions. The three authors have agreed on these modes not as a strict classifying system, but as a heuristic tool: We use this as the basis from which testable hypotheses can be derived.

Also, the four modes are not exclusive, and combinations of two or more modes were common. Also, alternative classifications were conceivable to us, both more coarse and more fine-grained.

These four modes represent something akin to “minimal pairs” in phonology (i.e., word pairs that are distinguished by just one single phoneme, such as “house–mouse and “pull–pool”): For phrase transitions of the morphing category “timbre,” *only* timbre is changed from pre- to post-transition, while pitch and temporal properties stay the same (and accordingly for the other three categories).

All modes of morphing are set apart from a class of high-contrast transitions. These are characterized by a *lack* of similarity between sounds/sequences before and after the transition.

#### Type 1: Timbre

**FIGURE 4:**
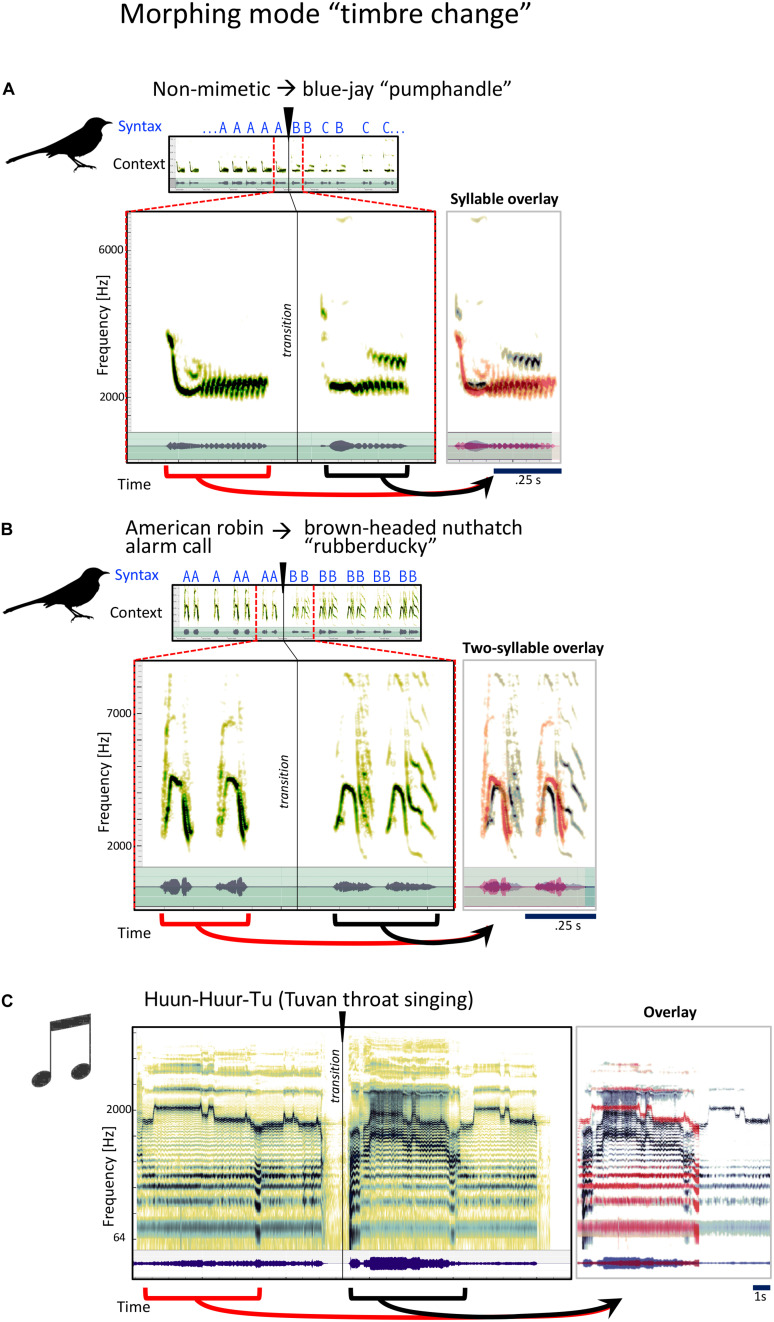
Examples for the mockingbird morphing mode “timbre change,” and example for a similar musical strategy. **(A,B)** Spectrograms and waveforms for three examples of phrase transitions representing “timbre” morphing, in which a phrase is followed by a phrase with similar syllables and temporal structure, the main difference being a modification in timbre. Small spectrograms on top show the song context with a clear transition from one song phrase to a new song phrase, which is also clearly evident when listening to the song files. Large spectrograms underneath zoom in to the section around the transition. Panels to the right show an overlay of the pre-transition (red) and post-transition (gray/black) syllables. Similarities between pre- and post-transition syllables become particularly clear in the overlay pictures. **(C)** Similar illustration for a musical example of Tuvan throat singing from the group Huun-Huur-Tu, in which a whistling phrase (pre-transition) is picked up again (post-transition) in a different timbre.

In this mode, most acoustic features of the syllable (syllable length, dynamic structure, pitch, etc.) stay similar, but the timbre, or tone quality changes. Since the mockingbird song is often based out of imitations of many different other species’ songs and calls, to accomplish this kind of transition, he often finds a sound from a completely different species that has the same rhythm, but a different texture or spectral character:

To the untrained human ear, the two phrases in Figure 4A sound remarkably similar. They are each from a series of several rhythmic repetitions of each sound, something characteristic of how the mockingbird uses his imitations. Gammon, with his years of listening experience, identifies the second sound as the blue jay’s “pumphandle” call. The bird gets there via a non-mimetic sound that sounds intriguingly similar to the pumphandle call: He basically “morphs” into his blue jay imitation. In the next example (Figure 4B), Gammon identifies the first sound as the alarm call of the American robin. The second is the “rubberducky” sound of the brown-headed nuthatch. Why does the mockingbird transition from the robin to the nuthatch? We do not know. But *sonically* we may identify a quality of morphing which we aim to show is one of the characteristic qualities of mockingbird song, to our knowledge, a quality that has not been examined before: Rhythm roughly constant, timbre changes.

Timbre change, however, is a common strategy in many kinds of human music. Here we see it used very clearly in a solo performance of Tuvan throat-singing by the group Huun-Huur-Tu (Figure 4C), where a single human voice changes the quality of the upper overtone partials so it sounds like more than one person is singing.

#### Type 2, Pitch Change

**FIGURE 5:**
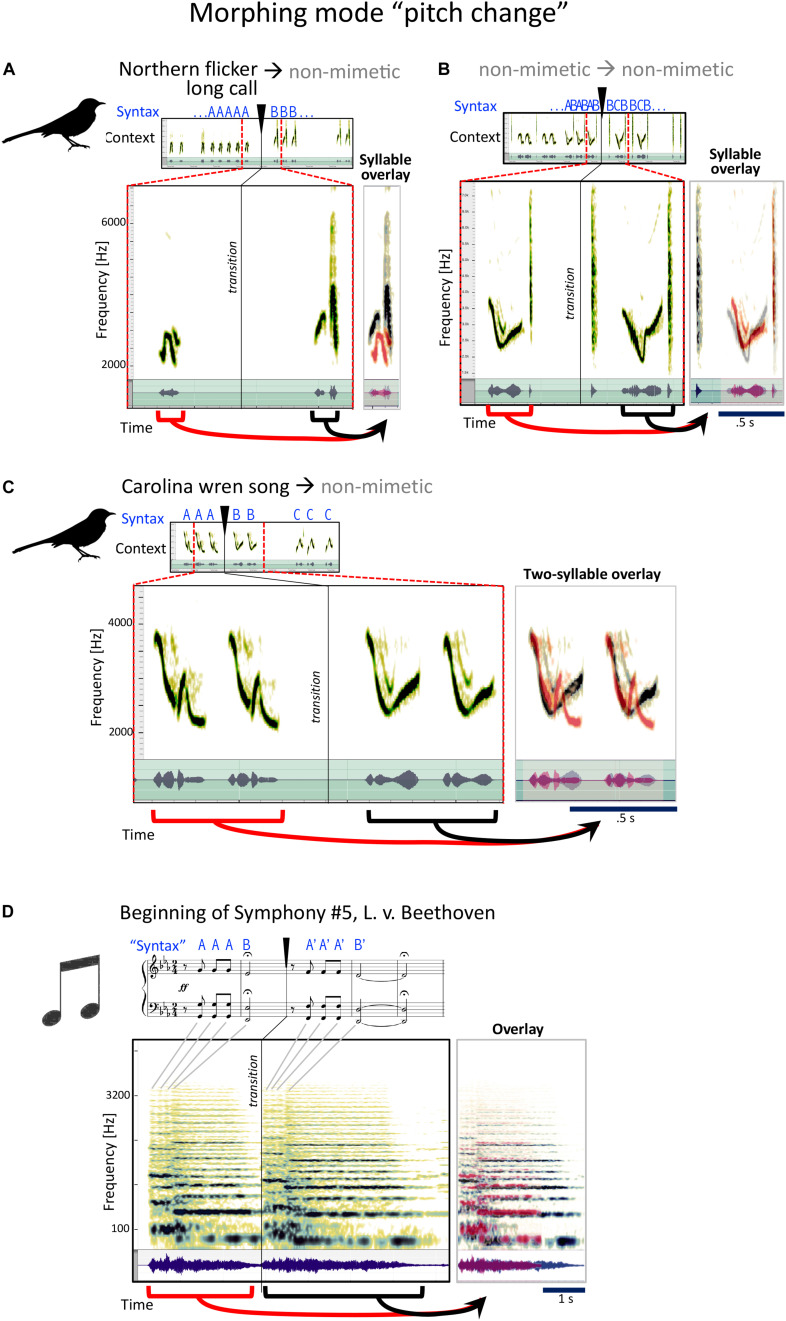
Examples for the mockingbird morphing mode “pitch change,” and example for a similar musical strategy. **(A–C)** Spectrograms and waveforms (as in Figure 4) for three examples of phrase transitions representing “pitch” morphing, in which a phrase is followed by a phrase with similar syllables, the main difference being a modification in pitch. **(D)** The beginning of Beethoven’s fifth symphony follows a similar strategy, with a four-note sequence repeated with a different pitch.

In this category (Figure 5), the primary quality noticed in the morph is a similar phrase, where the pitch or frequency is the most clearly changed quality. Pitch change could apply to the specific pitches used in Hz (Figure 5A), to the pitch contour (Figure 5B), or both. In Figure 5A, mockingbird A sings the long call of the Northern flicker and then takes it up a few notes with the same rhythm in a non-mimetic phrase. A little later, he combines a melodious whistle with a “chk” sound and then tries out another, even more melodious variant of that whistle with the same “chk” (Figure 5B).

Some minutes later, he moves from a Carolina wren call to a non-mimetic, connected by pitch morphing (Figure 5C).

This is probably the most familiar of the four types from human music. The beginning of Beethoven’s Fifth Symphony (Figure 5D), heard as quite radical in its time, is a clear example of this that most of us know well.

#### Type 3: Stretch, or Half-Time

**FIGURE 6:**
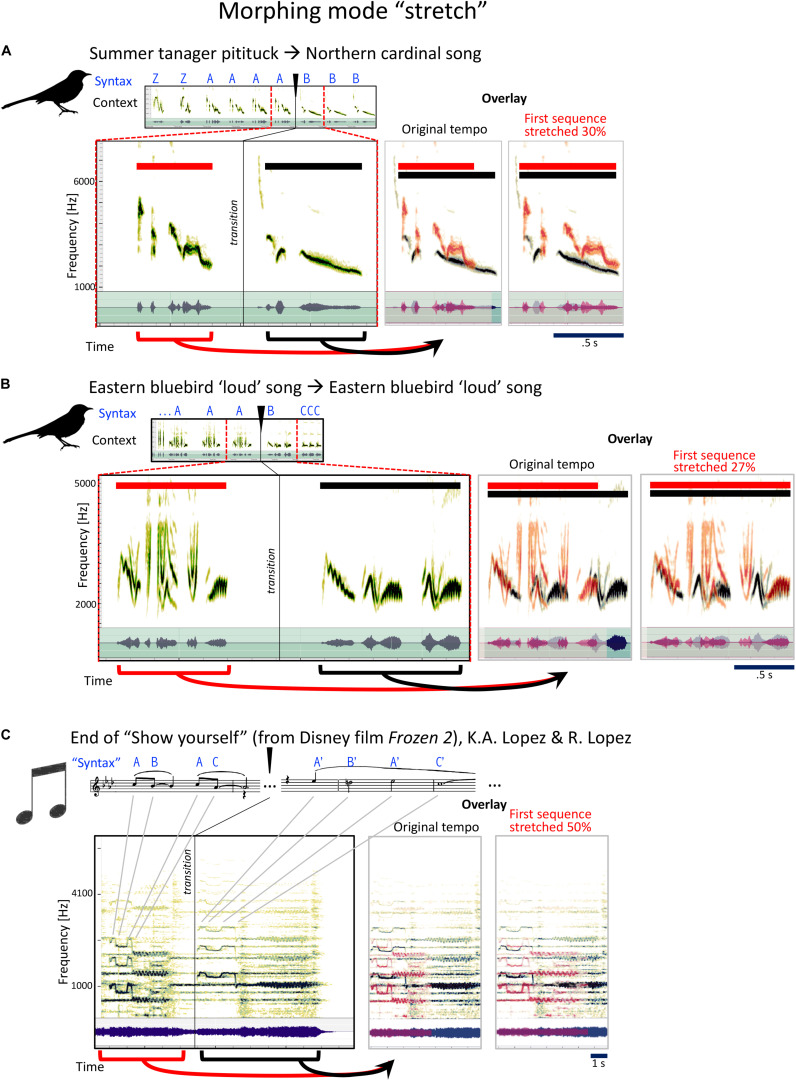
Examples for the mockingbird morphing mode “stretch,” and example for a similar musical strategy. **(A,B)** Spectrograms and waveforms (as in Figure 4) for two examples of phrase transitions representing “stretch” morphing, in which a phrase is followed by a similar, but slowed-down phrase. Note that both examples also include additional changes in timbre and/or pitch. **(C)** The end of the song “Show yourself” from the Disney film Frozen 2 follows a similar strategy, with a four-note sequence repeated at half the tempo (this example also includes a pitch change).

The third strategy commonly used by mockingbirds is to slooooow down, or stretch the syllables they are singing (Figure 6). Here, mockingbird A takes a summer tanager pitituck and stretches it out slower than half time into a fragment from a northern cardinal (Figure 6A):

Next is an extraordinary moment: the “loudsong” of the Eastern bluebird (Figure 6B), first at regular speed, then slowed down in an easy-to-hear decelerando, a relaxing of tempo from allegro to andante. A beautiful, musical movement. These are two related songs of the Eastern bluebird, the second much slower than the first. Figure 6C illustrates how the stretching technique is used by Idina Menzel toward the end of the song “Show Yourself” from the Disney movie *Frozen 2.*

#### Type 4: Squeeze

Very simply, this is the opposite of stretch. Take one phrase, and have the next phrase be similar, but much faster.

**FIGURE 7:**
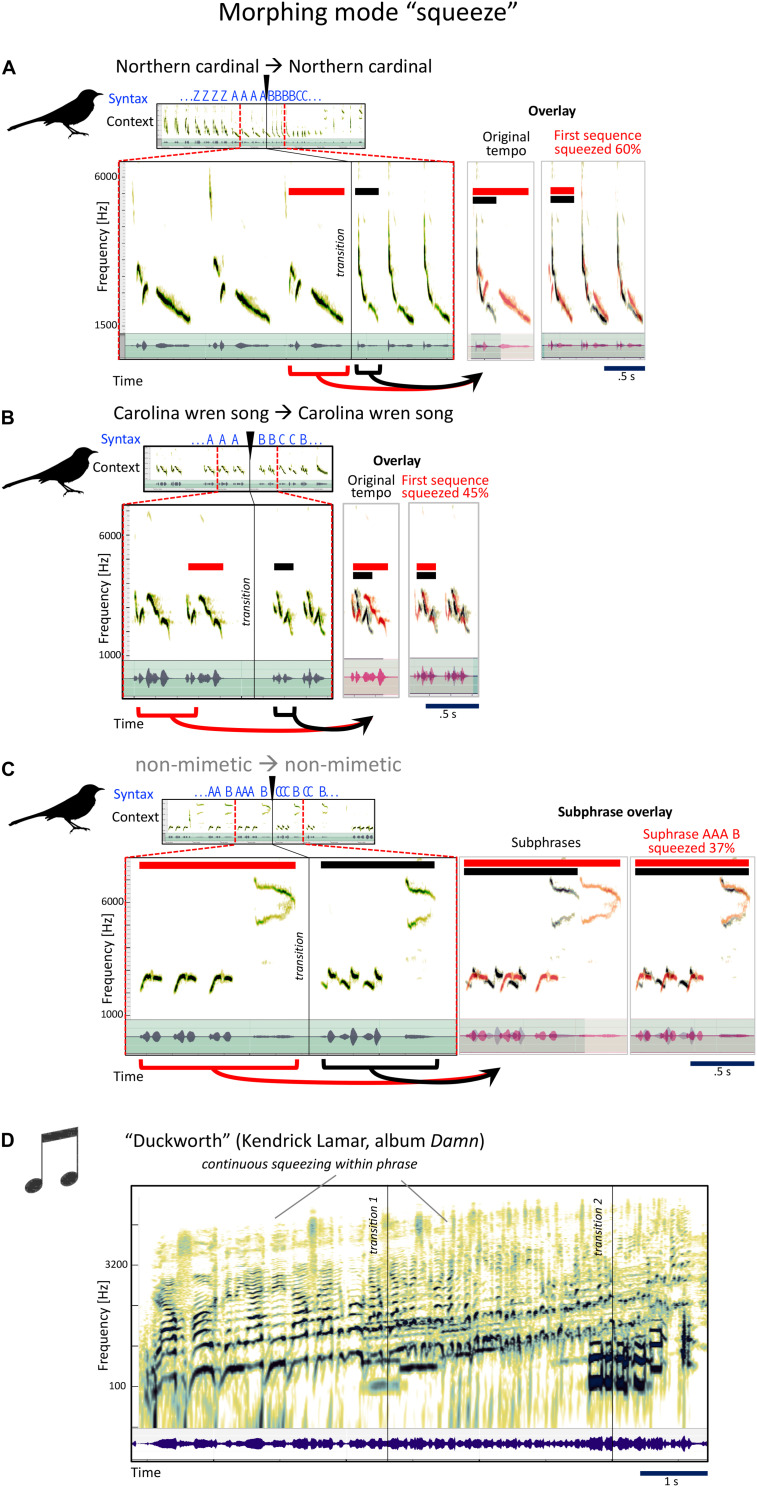
Examples for the mockingbird morphing mode “squeeze,” and example for a similar musical strategy. **(A–C)** Spectrograms and waveforms (as in Figure 4) for three examples of phrase transitions representing “squeeze” morphing, in which a phrase is followed by a similar, but sped-up phrase. **(D)** Kendrick Lamar’s song “Duckworth” contains a section that uses a similar strategy of speeding up (here, however, the acceleration is more continuous than in the mockingbird examples).

The pre- and post-transition *sounds* in Figures 7A,B have many acoustic aspects in common: contour, pitch, and timbre. The post-transition syllable is basically faster. Morphing tends to change one or a few aspects of the sound, not all. This one: Timbre/pitch constant, rhythm faster. Squeeze.

Later on, still in the same song, a phrase of uniquely syrinxian beauty (Figure 7C), where this single bird’s ability to make at least two highly contrasted sounds at the same time produces rhythmic and tonal relatedness, from three to two to three to six—four, interspersed by high antiphonal whistles. This image is so much like a musical score. While none of the sounds are imitations, Gammon says some of their elements are recycled from mockingbirds’ own functional calls, e.g., from fledglings’ begging calls and from calls used in other social contexts, like fighting or contact calls. Morphing mode: squeeze.

Once the four morphing modes have been identified, we find them often, sometimes clearly just one, other times combined with each other.

### Sequences of Morphs

**FIGURE 8:**
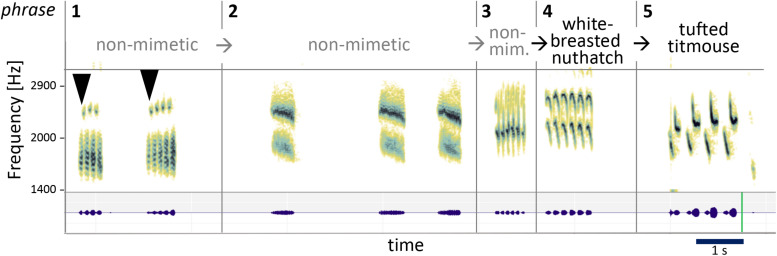
Example sequence of several morphs in a row. A train of syllables separated by longer pauses is repeated and morphed in several steps. From part 1 to part 2, syllables become denser and noisier, and pitch rises. From part 2 to 5, syllables are successively stretched, accompanied by slight pitch and timbre changes. This juxtaposition of elements seems to reflect sonic relationships, regardless of status of mimesis (the bird uses both non-mimetic and mimetic elements).

The composite example in Figure 8 shows several morphs in a row at work.

While the first three phrases are non-mimetic, the fourth is a white-breasted nuthatch and the fifth a tufted titmouse song. But besides their mimetic origins, they are clearly sonically related. Between each change, some acoustic aspects are altered and others stay the same. We will stay focused on the transitions: Onetwothreefour (first arrowhead). Onetwothreefourfive (second arrowhead). Syllable length half a second. Take the same length, rise the pitch, make it a steady noisier complex sound, and repeat three times. It is common for non-mimetic elements to keep similar “time lengths” before vs. after a transition—clear example of morphing (divided into 13, 14, 13 tiny tones if you stretch). Two repetitions of six more tonal each, each one stretched out in overall length again. Morphing modes: squeeze, stretch, stretch, stretch, with a bit of timbre change along the way. First he speeds up, then he stretches, streeeetches, streeeeeeeeeetches.

For an example from human music this is also a compound one, where squeezing is combined with pitch morphing from Kendrick Lamar’s album *Damn*, the song is “Duckworth,” the conclusion of the album (Figure 7C). This did not win the Pulitzer Prize in music composition for nothing… Lamar uses a whole series a compositional strategies that have worked wonders for mockingbirds for millions of years.

### Quantification of Morphing Type Occurrence in Three Birds: Morphing Seems to Be Common

**FIGURE 9:**
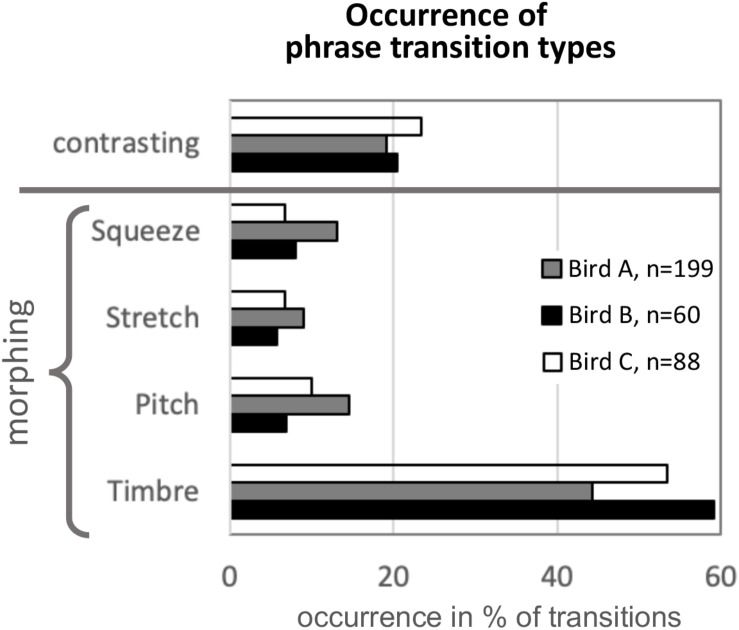
Occurrence of modes of morphing in song excerpts of three birds. Based on human auditory judgment (exploratory quantification by one observer), every phrase transition was assigned the most fitting morphing category, or the label “contrasting” if there was no salient morphing, i.e., low similarity between the pre- and post-transition phrase. Morphing mode “Timbre,” in which timbre is changed from pre- to post-transition while other syllable features stay mostly similar, was the most common, covering around 50% of all transitions. In only around 20% of transitions, pre- and post-transition syllables were dissimilar/contrasting. *n* indicates number of phrase transitions analyzed. Note that this analysis was based on a subset of phrases from birds A, B, and C (number of transitions analyzed are indicated in the legend).

Figure 9 shows how often the different morphing types occur in mockingbird song, based on categorization by human ear of a subset of data from birds A, B, and C (for the number of transitions analyzed, see the figure legend). Note that because the goal was merely exploratory, only one observer collected these data. He classified the transitions as either contrasting or morphed, and the morphed ones according to their most salient mode of morphing. Despite the small sample size, the different transition types as detected by one observer were surprisingly consistent across the three birds. Roughly half of all transitions were morphing based on timbre, and only in about 20% of phrase transitions, there was little acoustic similarity between pre- and post-transitional syllables (“contrasting” transitions, as opposed to morphing). Note that the *relative proportions* of the four morphing modes may have to be taken with a grain of salt, as “combo morphing” is common, and the high share of “timbre” morphing may be partly due to what humans happen to find most salient. However, the overall occurrence of morphing vs. contrasting patterns suggests that morphing is ubiquitous in the song (more than three times as common as contrasting transitions).

### Quantitative Pilot Analysis of Adjacent-Phrase Similarity: Is the Morphing Really Happening?

Is our strong subjective impression of morphing actually an acoustic property of mockingbird song—i.e., something that the birds actively do and control—or could it be an illusion, created by biases in the human auditory system? We conducted an analysis of acoustic similarity between adjacent phrase pairs vs. random phrase pairs, with the goal to collect quantitative evidence for (or against) morphing as a singing mode in mockingbirds. Our hypothesis is that the birds’ tendency for morphing results in higher acoustic similarity between adjacent as compared to distant phrases. We assessed acoustic similarity in terms of four acoustic features:

1. The syllable amplitude envelope, representing the syllable’s internal temporal structure, as apparent in *waveforms* in Figures 4–7.
2. The syllable frequency contour (representing pitch progression of a syllable, as apparent in the *spectrograms* in Figures 4–7).
3. The syllable Wiener entropy contour [a measure for tone- vs. noise-like-ness that would be high in a syllable that sounds like “fiuuuuu” (e.g., the last, whistle-like part of note “B” in Figure 6A) and low in a noisy syllable that sounds like “chk” (e.g., the broadband short note “B” in Figure 5B)].
4. Syllable duration.

To compare syllable amplitude, frequency, and entropy, we calculated *Euclidean distance* between the millisecond-wise time courses (high similarity between syllables means low Euclidean distance; see section “Materials and Methods” for details). Syllable durations were compared in relative terms, as *duration ratio* (shorter syllable’s duration: longer syllable’s duration; see section “Materials and Methods”).

Table 2 illustrates how each of the four modes of morphing would affect adjacent-phrase similarity/distance if our hypothesis of morphing as a true strategy is correct. Note that the presence of each of the morphing modes would increase similarity (decrease distance) between adjacent phrases. Since every transition is not characterized by morphing—*contrasting* transitions happen as well, and often—we do not expect the effect to be large. However, if morphing is not just an illusion of our own biased perceptual system, we should expect that it lowers adjacent-phrase Euclidean distances to *some* degree.

**FIGURE 10:**
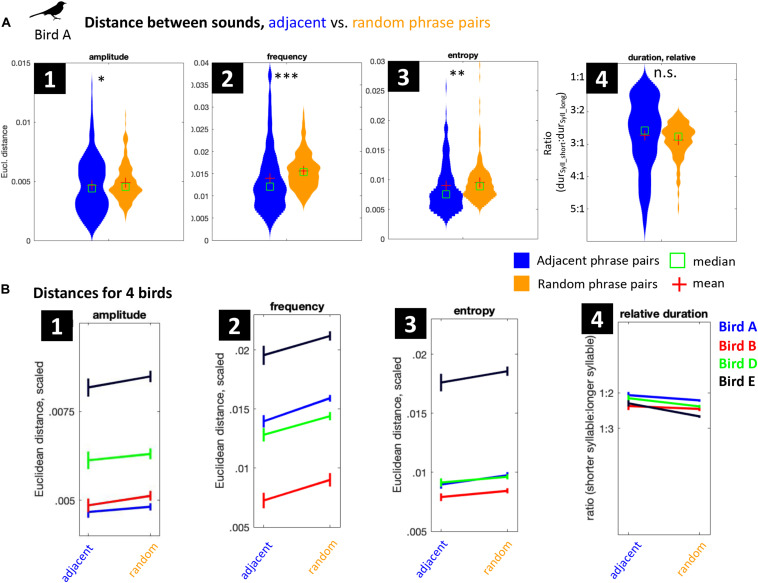
Acoustic syllable similarity between adjacent-phrase pairs vs. random-phrase pairs. **(A)** Four metrics for acoustic distance between syllables of adjacent (blue) vs. random distant (orange) phrases, for bird A. Sampling methods are illustrated in Figure 2. Mean Euclidean distances for (1) syllable amplitude (reflecting internal syllable dynamics), (2) frequency (reflecting syllable pitch), and (3) Wiener entropy (reflecting syllable timbre. (4) Syllable duration ratio (shorter syllable:longer syllable), reflecting whether the syllables are of similar or different length. Bird A’s amplitude, entropy, and frequency are significantly more similar between adjacent than between random phrases (Bonferroni-corrected for multiple comparisons). Syllable durations are not significantly different. **(B)** Same comparisons for four birds (including bird A, in blue). Error bars show SEM.

Figure 10A shows that for bird A, three out of four acoustic syllable features are indeed more similar between adjacent phrases than expected by chance (i.e., between random phrases). Bird A kept especially syllable frequency similar in adjacent phrases, but also internal syllable structure (amplitude) and syllable timbre (represented by Wiener entropy) are more similar than expected (for *p-*values, see Table 3). After Bonferroni correction for multiple comparisons, differences are significant for syllable amplitude, frequency, and entropy, but not for duration ratio. Birds B–D show a similar overall pattern (Figure 10B; see Table 3 for statistics). Together, this suggests that when producing a train of phrases, mockingbirds pick phrases with similar acoustic properties. When we hear them morphing between phrases, this is not just due to biases in our auditory system, but due to actually higher acoustic similarity between adjacent phrases.

**TABLE 3:** Statistics for adjacent-phrase vs. distant-phrase comparisons for birds A, B, D, and E.

	Amplitude	Frequency	Wiener entropy	Relative note duration
Bird A	***p* = 0.006**	***p* < 0.001**	***p* < 0.001**	*p* = 0.152
Bird B	***p* = 0.007**	***p* < 0.001**	***p* = 0.003**	***p* = 0.001**
Bird D	*p* = 0.091	***p* < 0.001**	*p* = 0.074	*p* = 0.044
Bird E	***p* = 0.012**	***p* < 0.001**	***p* = 0.005**	*p* = 0.041

*Bold font indicates statistical significance (t-test, Bonferroni corrected for multiple comparisons, see section Statistics).*

## Discussion

In this exploratory analysis of mockingbird song—a song that’s intriguing and beautiful to human ears—we approached our topic under a dual perspective: from a “musicological” angle, we first analyzed our own subjective listening experiences. Then, we took on a behavioral-quantitative perspective: based on our auditory impressions, we formulated a (testable) hypothesis about acoustic structure, namely, that syllable similarity of adjacent phrases should be higher than expected by chance. To collect quantitative evidence for (or against) this hypothesis, we analyzed the acoustic structure of mockingbird song in a quantitative way by measuring similarity of acoustic features between the syllables of adjacent vs. non-adjacent pairs of phrases.

The quantitative evidence in this exploratory analysis is based on a small sample, and we cannot claim to have tested our hypothesis in a thorough way. Nevertheless, the evidence we gain from analyzing four birds’ songs is telling: Syllables *are* more similar between pairs of adjacent than between random phrases in the song of four tested birds. This suggests that our subjective listening impressions of morphing do not only originate from biases in our auditory processing mechanisms, but that morphing is a part of mockingbird song that may be as salient to the birds themselves as it is to us. The hypothesis we have is that mockingbirds are inherently interested in the sonic relationships between a plethora of bird songs. They form their songs based on these four morphing modes, and find from among the other species’ songs they have heard phrases that follow their rules, like human composers following voice leading and rules of harmonic motion. But the birds do not use human rules, but those endemic to their own species, honed over the generations through sexual selection. Our analysis tries to make explicit those rules in human terms, to help answer the question, “What specifically in that song tells us it is the song of a mockingbird?”

Once we admit this structure, it is possible to suggest reasons such structure might have evolved. On the proximate level, morphing might be driven in part by the physical and physiological constraints associated with switching between song phrases. Producing acoustically-similar phrases requires fewer adjustments to the muscles of the syrinx (Podos and Nowicki, 2004; Zollinger and Suthers, 2004), as well as fewer adjustments to the underlying song nuclei within the brain (Sober et al., 2008). Morphing might therefore, in part, be a behavioral strategy shaped by proximate mechanisms that reduce the energetic demands of singing (Laughlin et al., 1998). While we cannot rule this out, we think that two aspects of mockingbird morphing make this explanation through energetic efficiency lacking: First, morphing also occurs between syllables that are not directly adjacent, but can be interspersed with other, often contrasting syllables, as in the examples in Figures 5B, 6B, 7C. In these examples, energetic benefits of low-contrast progressions are likely limited. Second, morphing takes on a broad range of different modes, as we detail in Figures 4–8. Sometimes, mainly pitch is changed; at other times, individual syllables in a repeated syllable-group are being replaced, while others are not. This means that the morphing can take place on higher-order levels in the hierarchical sequence, not only as a first-order relationship between adjacent syllables. We suspect that this distinguishes the mockingbird’s morphing from the more stereotyped, predictable acoustic morphing in many birds’ calls (Figures 1B,C)—which may be an interesting question for future research. But whether or not morphing sequences are favored by a particularly high efficiency of their neural or muscular control, their distribution suggests that there might be *some* kind of neural representation of spectral and temporal *similarity*. This aspect has not played a role in the research on neural control of birdsong as we are aware of.

On an ultimate level, the functional reasons behind morphing and other potentially aesthetic aspects of the mockingbird remain unclear. Darwin suggested all aesthetic aspects of birdsong result from sexual selection: The idea is that females prefer song with certain aesthetic features, and will procreate more likely with singers who produce those features, which makes them more pervasive within the entire species over time. Indeed, mockingbirds’ singing behavior is consistent with a strong role of sexual selection in shaping song structure: Although female mockingbirds will sing on rare occasions (unpublished data of Christine Stracey, and Gammon and Stracey, In Prep), the vast majority of song is produced by males and directed at females rather than other males (Merritt, 1985). The use of mockingbird song correlates strongly with whether a male is mated or not, and the timing of the reproductive state of his mate (Merritt, 1985; Logan and Hyatt, 1991; Wright, 2014). We also know that mockingbirds use more mimicry in their songs during the breeding season, particularly late in the breeding season when females are more physiologically exhausted and need more stimulation if they are going to be motivated to breed (Gammon, 2014). While the behavioral context of mockingbirds’ singing thus supports the notion of sexual selection shaping the song, it remains as yet unclear how any *specific* song feature may become a target of sexual selection. There have been many attempts to identify specific birdsong features that females prefer (like the famous “sexy syllables” of canaries, Vallet and Kreutzer, 1995). Such studies have often selectively focused on short, specific acoustic elements, ignoring the fact that they are part of complex songs. We suspect underlying this focus is the simplistic (and usually implicit) idea that individual acoustic elements have a fixed biological value recognizable to females and that the quality of an entire song could in turn be determined as some summary value of its elements. While a helpful heuristic for some research questions, this approach suffers from being unable to explain the structural complexity and inherent variability of birdsong: It would rather predict that the most successful song consists of endless repetitions of one or very few elements that have the best quality (like the bellowing of a stag), and adding any more, lower-quality elements should be strictly suppressed through sexual selection (Prum, 2017). When we are interested in a birdsong of high complexity and variability as the mockingbird’s, this model is therefore unlikely to further our understanding of song structure, evolution, or function(s). This is where adopting a slightly different heuristic—such as starting from our own listening experience to identify candidate structures that may be able to affect a listener’s interest or emotions—may help formulate more helpful research hypotheses (Rothenberg et al., 2014; Roeske et al., 2018). With this heuristic, we identified *morphing* as such a candidate strategy, or rule. Note that morphing is a considerably more abstract acoustic concept than elements like “sexy syllables” or features like trill tempo: it covers a broad range of *different* similarity-based relationships and operates on multiple hierarchical levels of the sound sequence.

In the case of mockingbird song, we as human listeners perceive as salient and attractive the complex acoustic phenomenon that we here termed “morphing.” It describes a strategy of similarity-based progression that can take on many different acoustic shapes: pitch-based transitions, syllable-structure-based transitions, timbre-based transitions, etc. Morphing thus describes an entire class of different and variegated acoustic phenomena, and thus resembles a rule rather than a particular acoustic element. Interestingly, this “rule” strongly overlaps with how we structure our own music. Where could this similarity come from? After all, sexual selection is less likely to play a major role in the cultural evolution of musical structure (Mosing et al., 2015; Bongard et al., 2019). This is a puzzling and important question for further research: If the evolution of mockingbird morphing and its human musical equivalents have been driven by similar forces, *sexual* selection is a poor explanation, since it applies to mockingbird song but not (or much less so) to music. Perhaps *other selection processes* that drive the cultural evolution of music can play an equivalent role to sexual selection in mockingbird song. In both cases, the proximate function of raising a listener’s interest and changing his or her emotional state, together with *some* selection process, might have led to similar acoustic strategies (Rothenberg et al., 2014).

Of course, it is entirely possible that alternatively, mockingbird morphing is strongly driven by the “sexual” part of sexual selection, and its overlap with music structures is more accidental than equivalent. This can be tested: For example, it would be enlightening to investigate whether morphing is more prominent when females are fertile, and whether females, but not other males, respond differently to song with a lot (as opposed to little) morphing in it.

Until such behavioral studies have been performed, there is a chance that studies like ours on the aesthetic aspects of mockingbird song might say more about humans than they say about the birds themselves. Acoustic structures that are most salient to us might not be the same ones that are most salient to the birds. Since mockingbirds’ own perception of the dynamics of song transformation remains unexplored as of yet, it is still an open question what acoustic structures sound intriguing to the birds. Investigations of behavioral, hormonal, or neural responses to playbacks are needed for a better understanding of this question. It would be naïve, however, for scientists or musicologists to assume that mockingbirds do not have an aesthetic sense until the relevant studies have been performed. Indeed, given the widespread evidence for birdsong aesthetics in other songbird species (Doolittle and Brum, 2012; Doolittle et al., 2014; Rothenberg et al., 2014; Janney et al., 2016; Taylor, 2017; Roeske et al., 2018; Rothenberg, 2019), it seems more likely that mockingbirds have an aesthetic sense. And it is conceivable that morphing is a common aesthetic strategy that may apply to many species: For instance, Black-capped chickadees are known to transpose pitch of their fee-bee song (Horn et al., 1992; Gammon and Baker, 2004); Field sparrows speed up their syllables (similar to the morphing mode “squeeze,” Nelson and Croner, 1991), and Canyon wrens slow their syllables down (as in morphing mode “stretch,” Benedict et al., 2013).

We admit that at the basis of our approach is a deeply anthropocentric assumption: that our human perceptual experiences may be similar to those of other species, possibly even where phenomena of high cognitive, social, and cultural complexity are concerned, as in music or language. Of course, this assumption needs to be questioned and tested for every case in which it is tentatively adopted (our article attempts to be an example for this). It is therefore crucial to make falsifiable predictions based on the assumption of similar experiences between species, and to test them. The alternative assumption that biologists have embraced more readily in the past has been the idea that other species (and sometimes even our own species) should, by default, be assumed to be automata whose subjectivity is of no interest for biology. We think that it is at our peril if we adopt this idea in the context of birdsong, in which sexual selection based on subjective preference judgments is known to be a crucial driver of acoustic structure.

## Conclusion

This paper has been written by three people deeply engaged in birdsong from quite distinct perspectives. The lead author is a neuroscientist steeped in the traditions of statistical analysis of animal signals endemic to much of the literature. The second author is a musician and philosopher who has been investigating the connections between music and nature for many years, actively combing the historical record and applying this knowledge to his own musical development. The third author is a field biologist who is a true connoisseur of mockingbird songs, having listened to and cataloged them in the wild for quite some time.

We would like to think that our study shows the value of combining different forms of human knowledge in the investigation of a single problem: When confronted with a complex mockingbird song, a musician will hear one thing, an ornithologist another, and a signal analyst something else. The most complete human knowledge of any natural phenomenon comes from combining distinct human forms of knowing—no one perspective negates the others. They are strongest when applied together. (Rothenberg, 2019).

Though it is easy enough for a human listener to distinguish a mockingbird from his relatives the catbird and the brown thrasher (Boughey and Thompson, 1975), or from other mimicking birds like the starling, the blue jay, or the yellow-breasted chat, no one has until now tried to qualify or quantify the specific compositional strategies used by this unique species of bird that are so similar to our own music.

Our paper elected just to look at transitions from one syllable to the next; we see this as a first step toward a more comprehensive analysis of the way mockingbird song is assembled. Simply counting the species a mockingbird imitates is not the most salient method to make sense of his song, and neither would be picking individual recurring elements and test whether females respond to them in specific ways. We need to learn more about how the birds move from one syllable to the next, and how they assemble groups of syllable phrases together in very stylized ways that present songs that are specifically characteristic of this species. It is this very specific characteristic, or original aesthetic sense as posited by Charles Darwin, that we ought to be able to articulate and identify in the song of this species.

To those readers who feel we have overstepped the bounds of science by hearing music in the mere functional sounds of a bird, we offer these wise words from the poet and Zen master Norman Fischer, from his book-length poem *Nature* (Fischer, 2021):

Science lacks humanity when it misses a sense of play and rhyme—when it forgets that eye and world are one and there is no knowledge only discussion; when it loses sight of humankind as Nature’s extrusion, Nature’s way of creating paradox, linguistic pleasure, and novel modes of distraction and destruction which were engraved, as potential, in the first molecule of rugged and ragged life; when it fails to see itself in every rock and tree and star.

## Data Availability Statement

The raw data supporting the conclusions of this article will be made available by the authors, without undue reservation.

## Ethics Statement

Ethical review and approval was not required for the animal study because we worked only on field recordings of wild birds.

## Author Contributions

All authors wrote this manuscript collaboratively on the basis of data collected by Dave Gammon.

## Conflict of Interest

The authors declare that the research was conducted in the absence of any commercial or financial relationships that could be construed as a potential conflict of interest.

## Footnotes

1 https://youtu.be/FwD0ij_CWoM

2 https://youtu.be/qEmOGU87lkY

3 https://www.xeno-canto.org/species/Mimus-polyglottos

4 http://soundanalysispro.com/matlab-sat

## Supplementary Material

Supplementary Audio 1 Mockingbird Morphing Music all figures audio.

Supplementary Audio 2 Bird C.

Supplementary Audio 3 Bird A.

Supplementary Audio 4 Bird B.

Supplementary Audio 5 Bird E.

Supplementary Audio 6 Bird D.
