# Supplementary material for: Mockingbird Morphing Music: Structured Transitions in a Complex Bird Song
Source: Front Psychol. 2021 May 4;12:630115. doi: 10.3389/fpsyg.2021.630115 (PMC8129044; doi:10.3389/fpsyg.2021.630115)
Supplement: Supplementary file 7 [file Data_Sheet_1.docx]

**Explanation of Supplementary Material**

for

*Mockingbird Morphing Music*

The audio data referenced in this paper is all included in the supplementary materials link, consisting of six audio files, which must be properly labelled as:

“Audio 1” is “Mockingbird Morphing Music all figures audio”

“Audio 2” is “Bird C”

“Audio 3” is “Bird A”

“Audio 4” is “Bird B”

“Audio 5” is “Bird E”

“Audio 6” is “Bird D”

These are the mockingbird recordings used in our analysis.

Tina C. Roeske

David Rothenberg

David E. Gammon
